# Supplementary material for: Divergent coronaviruses discovered in the virome of lamprey with reddening syndrome
Source: J Virol. 2026 Sep 2;100(9):e00645-26. doi: 10.1128/jvi.00645-26 (PMC13595959; doi:10.1128/jvi.00645-26)
Supplement: Supplementary file 1 [file jvi.00645-26-s0002.pdf]

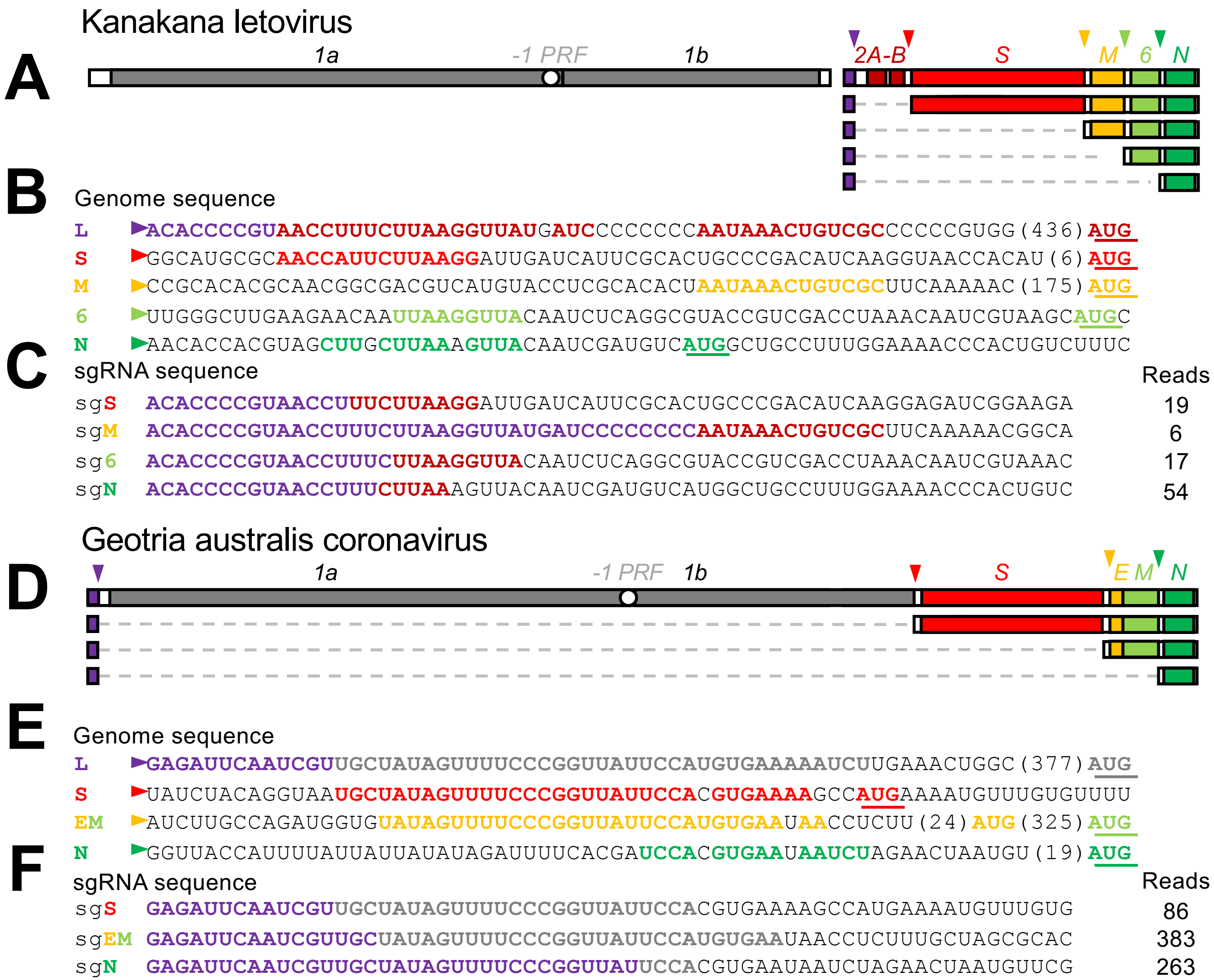

Supplementary Figure 2. Genome organization and evidence for discontinuously-synthesized subgenomic RNAs in novel lamprey coronaviruses. (A,D) Maps showing the reconstructed genome organization, color-coded genes and potential transcription-regulatory signals. Sequences joined through discontinuous RNA synthesis are indicated with a dashed line. (B,E) Genome regions corresponding to leader (purple) and potential body sequences, color-coded according to the genome map for each virus. Start codons are underlined, and number of nucleotides not displayed are given in parentheses. (C,F) Reads containing discontinuously-joined leader and body sequences are shown, with number of reads detected in each dataset. Reads marked as potential sgRNAs have at least 10 nt of unique leader sequence, and 10 nt of unique body sequence flanking the potential crossover region.
