# Supplementary material for: Divergent coronaviruses discovered in the virome of lamprey with reddening syndrome
Source: J Virol. 2026 Sep 2;100(9):e00645-26. doi: 10.1128/jvi.00645-26 (PMC13595959; doi:10.1128/jvi.00645-26)
Supplement: Supplementary file 2 [file jvi.00645-26-s0001.docx]

**Supplementary Figure 1**. Evidence for biological connection between replicase and structural gene regions of a novel non-segmented coronavirus, but not segmented coronavirus. (A) Maps showing the reconstructed genome organization (top) and subgenomic RNA organization (below), with areas of analysis marked in red dashed boxes. (B) Results of BLAST search for reads mapping to regions indicated in panel A (hits), and their corresponding paired-end reads (paired ends). Hits and paired ends were then mapped back to the genome, and categorized as matching exclusively one segment or region, both, or neither. Reads marked as potential sgRNAs have at least 10 nt of unique leader sequence, and 10 nt of unique body sequence flanking the potential crossover region.

^aFor this analysis, the unsegmented Geotria australis coronavirus genome was separated into 2 regions at the stop codon of pp1ab, to simulate the segmentation of Kanakana letovirus.^

^bCapped at 1000 hits.^

^cReads mapping to the 5’-most 41 nt, found on both segment 1 and 2.^

^dIncluding some reads with leader-body junctions characteristic of coronavirus subgenomic RNAs.^

Segment1

Segment2

Kanakana letovirus

sgS

sgM

sg6

sgN

Region2

Region1

Geotria australis coronavirus

sgS

sgEM

sgN

A

B
